# Global landscape of SARS-CoV-2 mutations and conserved regions

**DOI:** 10.1186/s12967-023-03996-w

**Published:** 2023-02-25

**Authors:** Mohammad Hadi Abbasian, Mohammadamin Mahmanzar, Karim Rahimian, Bahar Mahdavi, Samaneh Tokhanbigli, Bahman Moradi, Mahsa Mollapour Sisakht, Youping Deng

**Affiliations:** 1 Department of Medical Genetics, National Institute for Genetic Engineering and Biotechnology, Tehran, Iran; 2 Department of Quantitative Health Sciences, John A. Burns School of Medicine, University of Hawaii at Manoa, Honolulu, HI 96813 USA; 3 Department of Bioinformatics, Institute of Biochemistry and Biophysics (IBB), University of Tehran, Tehran, Iran; 4 Department of Computer Science, Tarbiat Modares University, Tehran, Iran; 5 Discipline of Pharmacy, Graduate School of Health, University of Technology Sydney, Sydney, Australia; 6 Department of Biology, Faculty of Sciences, Shahid Bahonar University of Kerman, Kerman, Iran; 7 Department of Biochemistry, Erasmus University Medical Center, 2040, 3000 CA Rotterdam, The Netherlands

**Keywords:** SARS-CoV-2, COVID-19, Emerging variants, Genome, Amino Acid, Vaccines

## Abstract

**Background:**

At the end of December 2019, a novel strain of Severe Acute Respiratory Syndrome Coronavirus 2 (SARS-CoV-2) disease (COVID-19) has been identified in Wuhan, a central city in China, and then spread to every corner of the globe. As of October 8, 2022, the total number of COVID-19 cases had reached over 621 million worldwide, with more than 6.56 million confirmed deaths. Since SARS-CoV-2 genome sequences change due to mutation and recombination, it is pivotal to surveil emerging variants and monitor changes for improving pandemic management.

**Methods:**

10,287,271 SARS-CoV-2 genome sequence samples were downloaded in FASTA format from the GISAID databases from February 24, 2020, to April 2022. Python programming language (version 3.8.0) software was utilized to process FASTA files to identify variants and sequence conservation. The NCBI RefSeq SARS-CoV-2 genome (accession no. NC_045512.2) was considered as the reference sequence.

**Results:**

Six mutations had more than 50% frequency in global SARS-CoV-2. These mutations include the P323L (99.3%) in NSP12, D614G (97.6) in S, the T492I (70.4) in NSP4, R203M (62.8%) in N, T60A (61.4%) in Orf9b, and P1228L (50.0%) in NSP3. In the SARS-CoV-2 genome, no mutation was observed in more than 90% of nsp11, nsp7, nsp10, nsp9, nsp8, and nsp16 regions. On the other hand, N, nsp3, S, nsp4, nsp12, and M had the maximum rate of mutations. In the S protein, the highest mutation frequency was observed in aa 508–635(0.77%) and aa 381–508 (0.43%). The highest frequency of mutation was observed in aa 66–88 (2.19%), aa 7–14, and aa 164–246 (2.92%) in M, E, and N proteins, respectively.

**Conclusion:**

Therefore, monitoring SARS-CoV-2 proteomic changes and detecting hot spots mutations and conserved regions could be applied to improve the SARS‐CoV‐2 diagnostic efficiency and design safe and effective vaccines against emerging variants.

**Supplementary Information:**

The online version contains supplementary material available at 10.1186/s12967-023-03996-w.

## Introduction

Over the last two decades, we have seen three lethal coronavirus outbreaks, severe acute respiratory syndrome (SARS, 2002–03) (1), Middle East respiratory syndrome (MERS, since 2012) (2), and now coronavirus disease 2019 (COVID-19, since late 2019) (3). The ecological realities assume that coronaviruses continue to pose a potentially existential threat in the future (4, 5) and already have a significant health, social, and economic impact on millions of people globally. Severe acute respiratory syndrome coronavirus 2 (SARS-CoV-2), which causes COVID-19, appeared in early December 2019 in Wuhan, a city of 11 million populations in China’s Hubei province [90, 92]. According to the World Health Organization (WHO), more than 621 million individuals worldwide have been infected with SARS-CoV-2 during the COVID-19 pandemic as of September 2022 [1].

**Fig. 1:**
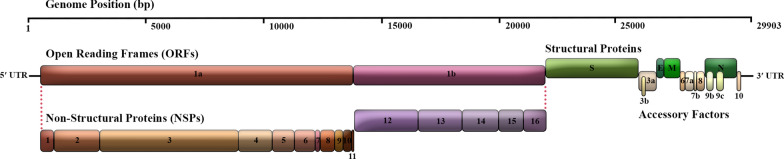
Schematic view of the SARS-CoV-2 genome arrangement. SARS-CoV-2 is an enveloped single-stranded positive-sense RNA beta coronavirus with a polycistronic genome ~ 30 kb in length. SARS-CoV-2 genome encodes several non-structural proteins (ORF1a and ORF1b, that are processed into NSP1-16) at the 5′-end, in addition to structural proteins (S, E, M, and N), and multiple other accessory proteins (ORF3a, 6, 7a, 7b, 8, 9b, 9c and 10) at the 3′-end

The SARS-CoV-2 is a lipid-enveloped, single-stranded, and positive-sense RNA (+ ssRNA) virus with a large genome length of 29,903 nucleotides that contains a 5′-cap structure, a 3′ poly(A) tail, 2 flanking untranslated regions (UTRs), and multiple open reading frames (ORFs) which encoding viral structural proteins and regulatory elements [14, 15] (Fig. 1). SARS-CoV-2’s gene content, function, and interactions with host factors have not yet been fully elucidated, notwithstanding its critical medical concern. Therefore, it is essential to have an insight into the basic virology of SARS-CoV-2 to develop better and more efficient therapeutics.

Approximately two-thirds of the entire genome of SARS-CoV-2 contains the ORF1a/b coding region, which is considered the largest ORF at the 5′ terminus. The -1 ribosomal frameshift between ORF1a and ORF1b leads to the formation of two co-terminal polypeptide domains called pp1a and pp1ab. The pp1a protein encoded by ORF1a is proteolytically cleaved into 11 mature non-structural proteins (NSP1-11). In contrast, the pp1ab protein expressed by ORF1ab is processed into 15 NSPs (NSP1-10 and NSP12-16) [90, 92]. The remaining third of the downstream genome near the 3′-terminus comprises ORFs encoding structural proteins (SPs) and accessory proteins [31]. Accessory proteins include ORFs 3a, 3b, 3c, 3d, 6, 7a, 7b, 8a, 8b, 9b, 9c, and ORF10 [32], which are distributed among the four major structural protein genes, namely spike surface glycoprotein (S), an envelope protein (E), membrane glycoprotein (M), and nucleocapsid phosphoprotein (N) [90, 92]. In addition to genomic RNA, several canonical subgenomic (sg) mRNAs are also produced [25, 34] (Fig. 1).

Numerous functional and structural domains of NSPs are well defined, including NSP3 as a papain-like protease (PL2pro) [40] and NSP5 as a 3C-like protease (3CLpro or Mpro) [84, 99, 100] that cleaves pp1a and pp1ab polypeptides in 15 NSPs [36]. NSP12, as a multi-subunit RNA-dependent RNA polymerase (RdRp) [95] in complex with NSP7 and NSP8 as co-factors, forms a replicase complex for replication and transcription of viral genomic RNA [38]. The nidovirus RdRp-associated nucleotidyltransferase (NiRAN) domain featured in NSP12 possesses a β-hairpin domain at its N-terminus [23]. NSP13 as a helicase (Hel) [74] and NSP14 as a proofreading exoribonuclease (ExoN) [44] are critical enzymes that facilitate viral RNA replication and transcription. Other NSPs are almost considered in the host cell and immune suppression. Structural proteins of the 3′-end are involved in viral interaction with the host cell angiotensin-converting enzyme 2 (ACE2) receptor, membrane fusion and entry of the virus into the host cells [63, 87, 89], viral assembly, morphogenesis, and release of virion particles from the host cell [43]. The function and expression of accessory proteins are still largely unknown.

Considerable genomes from all parts of the world have been sequenced and are available at the Global Initiative on Sharing All Influenza Data (GISAID; https://platform.gisaid.org/) [21, 35, 75] and NCBI (https://www.ncb.nlm.nih.gov/) from the onset of the pandemic in January 2020.

In this study, we used bioinformatics tools to process these massive datasets efficiently and evaluate approximately 10,300,000 SARS-CoV-2 genome sequences worldwide until April 28, 2022. In our high-throughput experiments, we tracked a systematic gene-by-gene comparison analysis with a reference genome (the first sequence data of a patient from Wuhan in the National Center for Biotechnology Information (NCBI) annotation NC_045512.2) to evaluate conserved genomic regions of SARS-CoV-2. Our study results provide a systematic resource to identify novel sequence features or functional elements worth consideration as vaccine candidates and therapeutic development.

## Methods

### Sequence retrieval

**Fig. 2:**
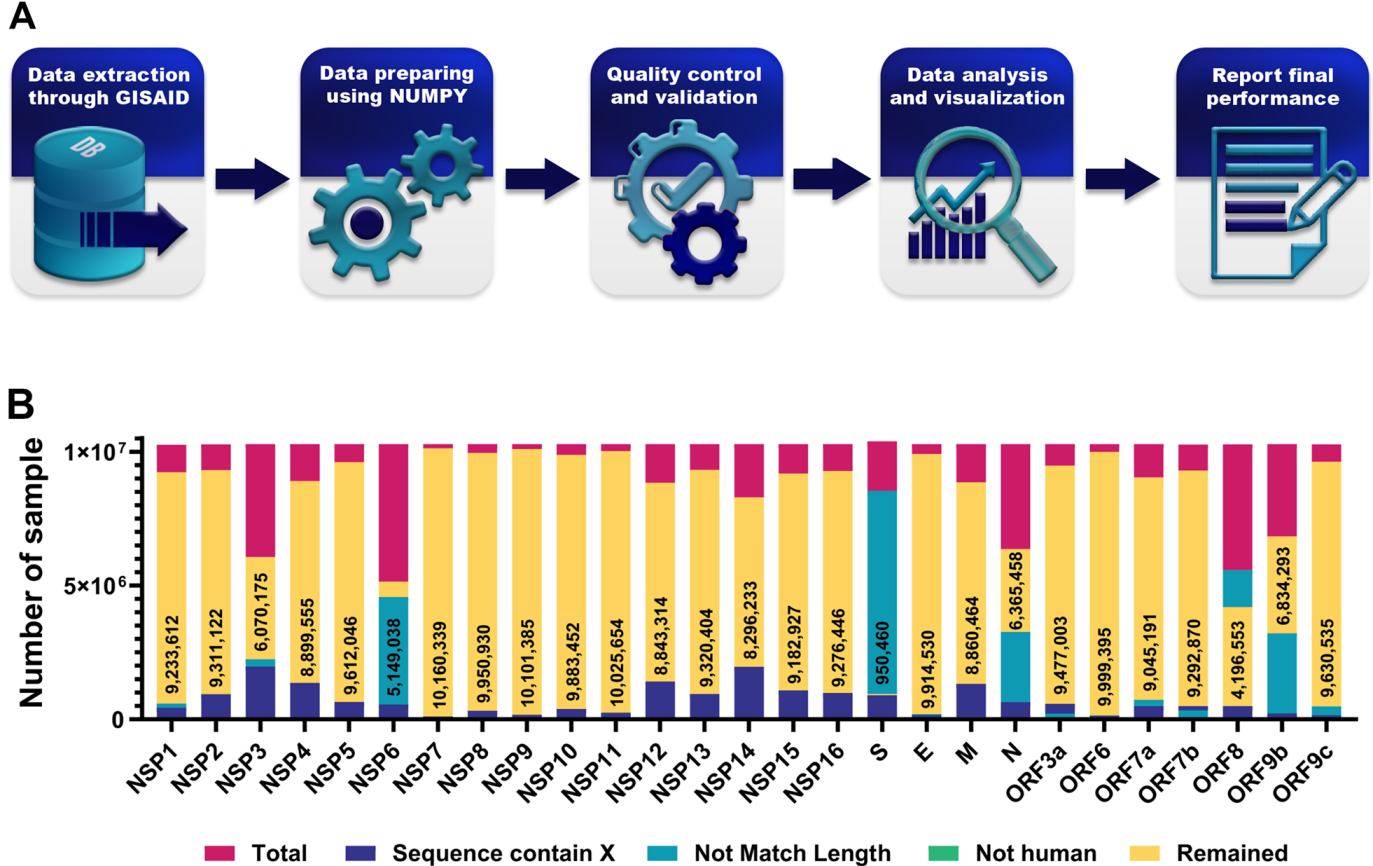
Overview of the study design. **A** Schematic describing the workflow of the study. **B** Illustration showing the number of SARS-CoV-2 samples. The number of analyzed SARS-CoV-2 samples are mentioned in bars

Figure 2 illustrates the research methodology workflow of our study. We obtained credentials to access data in the GISAID database (26–28) with Erasmus Medical Center authorization. The NCBI RefSeq SARS-CoV-2 genome (accession no. NC_045512.2) was considered as the reference sequence. The whole available data of SARS-CoV-2 full-length genome sequences (10,287,271 samples) and their amino acid sequences, including their geographical locations and submission dates of sample annotations, were downloaded in FASTA format from the GISAID databases by April 28, 2022.

### Sequence alignment and trimming

Python programming language (version 3.8.0) software was utilized for Pre- and post-processing FASTA files. The entire collected 10,287,271 sample sequences were aligned to the reference sequence using the EV couplings Python package. Excluding the 3ʹ and 5ʹ terminus regions was carried out after alignment to eliminate large numbers of missing and ambiguous reads and to achieve better alignment accuracy. Then excessively divergent, short, or long sequences, gaps (including dash and space characters), ambiguous nucleotides (such as N, B, and W), or non-specified amino acids (indicated by X) were removed. Eventually, genomes were filtered from non-human host species (such as mammals and birds) since no significant numbers existed to participate in the study Additional file 1.

### Sequence analyses and processing

After the trimming and filtering step, each of the remaining high-quality and high-coverage SARS-CoV-2 complete genome sequences was aligned against the reference sequence to identify variants, sequence conservation, and annotate them through the X functions of the Y package. Compared with the reference sequence, each amino acid-changing replacement in samples was defined as a variant. We recognized that as a conserved if there were no intergenic amino acid-changing replacements in the alignment. In such cases, their location of them was reported. The entire high-level processing was optimized using NumPy (version 1.16.2) and Pandas (version 0.22.0) python libraries Additional file 2.

After extracting conserved and hot spot regions, each sample’s continent name and geographic coordinates were detailed through pycountry-convert and titlecase Python libraries and displayed in global maps using Matlab programming language (version R2021a) software; Geobubble package. A flowchart outlining the whole procedure in this study is shown in Fig. 2.

### In silico analysis of mutations for prediction of protein stability

We have performed four different structure-based bioinformatics tools to examine the effect of several important mutations identified in this study on the stability of SARS-CoV-2 proteins. Different types of computational methods were developed to predict stability changes upon mutation. These methods estimate Gibbs free energy values (ΔΔG) to classify the effect of each mutation as stabilizing or destabilizing on the protein structure. DynaMut [68], DynaMut2 [69], MAESTROweb [39], and SDM [60] are web servers that are able to predict the effects of missense mutations on protein stability. The crystal structures of SARS-CoV-2 wild-type proteins were retrieved from Protein Data Bank (PDB) [8].

### Statistical analysis

Exploration, normalization, and visualization of the data were conducted using GraphPad Prism (version 8.0.2) and Microsoft Power BI software as data analytics tools. Clusters were identified through hierarchical clustering analysis using the stats library in the R (version 4.1.3) programming language.

## Results and discussion

### Top mutations in SARS-CoV-2 in different geographic areas

The mutation is the engine of evolution that generates genetic diversity. It has been demonstrated that mutations in the SARS-CoV-2 genome are responsible for a drastic change in the protein structure and lead to an increase in the infectivity, fitness, and virulence of SARS-CoV-2 [19]. 10,287,271 SARS-CoV-2 samples from February 24, 2020, to April 2022 were downloaded from the GISAID database. High-quality SARS-CoV-2 complete genome sequences for each gene were analyzed (Fig. 2). Mutational Analysis of the SARS-CoV-2 revealed a high rate of recurrent mutations across all regions (Table 1). Six mutations had more than 50% frequency in global SARS-CoV-2. These mutations include the P323L (99.3%) in NSP12, D614G (97.6) in S, the T492I (70.4) in NSP4, R203M (62.8%) in N, T60A (61.4%) in Orf9b, and P1228L (50.0%) in NSP3. Interestingly, the frequency of these top mutations was higher in North America and Europe than in South Africa and Oceania (Table 1).

**Table 1:** Most common mutations in different SARS-CoV-2 genes

SARS-CoV-2 region	Mutation	Worldwide (%)	Africa(%)	Asia(%)	Europe(%)	North America(%)	South America(%)	Oceania(%)
NSP1	S135R	8.01	3.49	8.10	12.5	2.24	0.41	12.2
NSP2	T85I	3.94	15.6	1.79	0.64	9.27	1.05	1.48
NSP3	P1228L	50.0	32.2	45.1	49.0	53.6	44.1	50.1
NSP4	T492I	70.4	52.6	60.5	73.3	69.4	59.8	77.9
NSP5	P1328	30.8	23.7	25.3	34.6	27.2	39.2	21.9
NSP6	T77A	71.1	50.0	57.2	78.2	66.8	70.3	65.2
NSP7	L71F	0.22	0.14	0.25	0.13	0.20	2.8	0.03
NSP8	Q24R	0.70	0.18	0.06	0.44	1.29	0.12	0.03
NSP9	T35I	0.50	0.33	0.13	0.37	0.80	0.18	0.03
NSP10	T102I	0.16	0.05	0.07	0.17	0.19	0.10	0.05
NSP11	S6L	0.16	0.27	0.08	0.16	0.19	0.10	0.04
NSP12	P323L	99.3	95.8	98.7	99.5	99.3	99.5	97.7
NSP13	P77L	42.6	35.1	44.6	40.0	46.7	32.2	39.2
NSP14	A394V	35.1	22.5	31.7	34.9	37.0	24.9	36.5
NSP15	T112I	8.97	5.36	10.1	13.6	2.56	1.44	12.4
NSP16	R216C	1.26	1.97	0.08	0.09	3.36	0.07	0.12
Spike	D614G	97.6	91.2	95.3	97.5	98.3	99.5	90.1
Orf3a	S26L	43.1	34.4	46.0	40.5	47.2	32.8	37.0
Envelope	T9I	30.6	21.9	26.5	35.0	25.7	21.3	38.8
Membrane	I82T	47.1	42.4	47.8	45.0	52.1	34.1	40.6
Orf6	D61L	9.49	4.82	10.1	14.6	2.70	0.58	13.0
Orf7a	T120I	41.4	33.3	43.9	39.4	45.3	31.2	10.5
Orf7b	T40I	39.5	30.6	33.2	39.2	41.7	33.8	40.6
Orf8a	S24L	3.53	0.14	0.09	0.09	9.42	0.05	0.44
N	D377Y	63.2	48.8	60.4	63.2	65.6	41.7	–
Orf9b	T60A	61.4	45.1	59.1	61.7	63.4	41.0	59.3
Orf9c	G50N	48.8	37.9	47.4	52.8	42.4	–	51.02

### Mutation rate in the SARS-CoV-2 genome

The mutation rate is the value at which the base pair or a larger region of DNA changes are made during the genome replication. Understanding the rate of mutation is of fundamental importance because the rate of mutations has explained the emergence of new SARS-CoV-2 variants and their establishment in natural populations.

The mutational profile of SARS-CoV-2 genome sequences has been changed during pandemics. Therefore, genomic surveillance of SARS-CoV-2 is critical to monitoring SARSCoV-2 genetic variability, which can improve diagnostic tools, vaccines, and immunotherapeutic interventions against COVID-19 [16]. RNA viruses vary in mutation rates, such as 1.35 × 10–5 in Influenza H3N2 and −1 4 × 10−5 mutations per target in HIV [27, 51]. The SARS coronavirus mutation rate is calculated at 9.0 × 10−7 mutations per nucleotide per replication cycle (m/n/rc) which is lower than most RNA viruses [20].The average mutation rate of 4 × 10^−4^ nucleotide substitutions/site/year [71]. RNA proofreading capability of Coronavirus preserves its genome since previous studies reported that nsp14 acts as a 3′-5′ exoribonuclease that explains the coronaviruses' extraordinary length single-stranded linear genome [53, 76]. The mutation rate of the SARS-CoV-2 genome has thus been estimated at 1 × 10−3 substitutions per base (30 nucleotides/genome) per year under neutral genetic drift conditions [85] or 1 × 10^−5^–1 × 10^−4^ substitutions per base in each transmission events [86]. Li et al. analyzed the evolution rate of SARS-CoV-2 32 genomes of virus strains between December 24, 2019, and January 23, 2020. The mean evolutionary rate for SARS-CoV-2 32 genomes ranged from 1.7926 × 10^−3^ to 1.8266 × 10^−3^ substitutions per site per year [41, 42]. Four months after the pandemic, the mutation rate for a complete SARS-CoV-2 genome with 29,903 nucleotides was 3.95 × 10−4 per nucleotide per year. The SARS-CoV-2 without its non-structural proteins 13 to 16 (Nsp13-Nsp16) exhibits an unusually high mutation rate [73]. This rate is lower than other RNA viruses, such as influenza A/H3N2 (10.9 × 10^−6^ nucleotide substitutions per site per day) [57]. Motayo et al. reported that the evolutionary rate of the Afr-SARS-CoV-2 from February 24 to April 24 was 4.133 × 10^−4^ substitutions/site/year [54].

The number of studied different sequences of the SARS-CoV-2 proteins is summarized in the Fig. 2. The Analysis of the worldwide SARS-CoV-2 genome revealed that no mutation was observed in more than 90% of nsp11, nsp7, nsp10, nsp9, nsp8, and nsp16 regions. (Fig. 2). Therefore, these regions of SARS-CoV-2 could be considered potential targets for diagnostics, treatment, or vaccine development.

According to our results, 99.72% of Nsp11 protein worldwide (from 99.61% in Africa to 99.93% in Oceania) did not illustrate any mutation. The independent function of NSP11 has not been characterized yet; however, NSP11 contributes to the interaction between the SARS-CoV-2 and host cell membrane [98]. Kaushal et al. analyzed the rate of mutation accumulation between January 19 to April 15, 2020, in the USA SARS-CoV-2 genome. They also found that NSP11 did not accumulate any mutation [33]. Saha et al. analyzed 198 Bangladesh-originated SARS-CoV-2 genomic sequences over 13 weeks. They found that nsp11 did not accumulate any mutation [70].

In the Nsp7 protein, 98.41% of aa sequences (from 95.65% in South America to 99.38% in Oceania) did not accumulate any mutation. Nsp7 forms a supercomplex with nsp8 and nsp12 and participates in coronavirus RdRP machinery that mediates SARS-CoV-2 replication [10]. Previous studies showed that the binding site for the nsp7-nsp8 heterodimer is well conserved, and the high conservation of nsp7 and nsp8 in coronaviruses proposed that nsp7-nsp8 heterodimer is a general component for all coronaviruses [97]. These results were also found in the USA SARS-CoV-2 genome [33]. mutant nsp7 proteins are significantly associated with mutant RdRp and could change the fidelity of genome replication [65].

In our study, 97.96% of Nsp10 protein (from 97.67% in South America to 98.59% in Africa) and 95.87% of nsp9 protein (from 97.67% in South America to 98.59% in Africa) in the world did not accumulate any mutation. NSP10 is encoded by ORF1a/1b, which comprises the RNA-synthesizing machinery of SARS-CoV2. Previous studies proved that nsp10 interacts with nsp14 and forms the NSP10–NSP14 complex, and this complex is critical for the viral replication process. Anand et al. studied nsp10 had the highest conservation thresholds, and molecular dynamics simulations revealed that the drugs Darifenacin, Nebivolol, Bictegravir, Alvimopan, and Irbesartan is targeted in this nsp. Nsp9 is a highly conserved region in beta coronaviruses and mediates viral replication, overall virulence, and viral genomic RNA reproduction. Saha et al. analyzed 198 Bangladesh-originated SARS-CoV-2 genomic sequences over 13 weeks. They found that nsp9 did not accumulate any mutation [70]. In the study of Kaushal et al. in the USA SARS-CoV-2 genome, no mutations were found [33].

In the nsp8, 95.52% of regions had no mutation (from 85.65 in South America to 98.12 in Oceania). In addition, 90.74% of Nsp16 protein in the world samples (from 61.97% in Oceania to 93.17% in Europe) did not accumulate any mutation. NSP8 is another component of the replication-transcription complex (RTC) in SARS-coronavirus [80]. In the cryoEM structure consisting of nsp7, nsp8, nsp12, and nsp13, the Interface domain is packed against nsp8 [14]. A previous study showed that SARS-CoV nsp8 was a mandatory de novo initiating RNA polymerase [80]. The viral RNA capping machinery protects mRNA from degradation by 5′ exoribonucleases, ensures efficient mRNA translation, and prevents recognition of viral RNA via innate immunity mechanisms. 2′-o-methyltransferase (2′-o-MTase) capping machinery was first discovered in the feline Coronavirus (FCoV) nsp16 [18].

In the worldwide SARS-CoV-2 samples, 97.39% of orf9c, 82.88% of Nsp6, and nsp14 76.01% had at least one, two, or three mutations. The nsp16 protein is an RNA cap modifying enzyme only active in the presence of its activating partner nsp10. In the SARS-CoV-2, Nsp16 is the leading enzymatic partner of the Nsp10-Nsp16 complex and protects from the host's innate immune recognition [45]. Identifying the 2′-O-MTase signature sequence in the SARS-CoV genome added nsp16 to the list of putative targets for antiviral drugs [77].

**Fig. 3:**
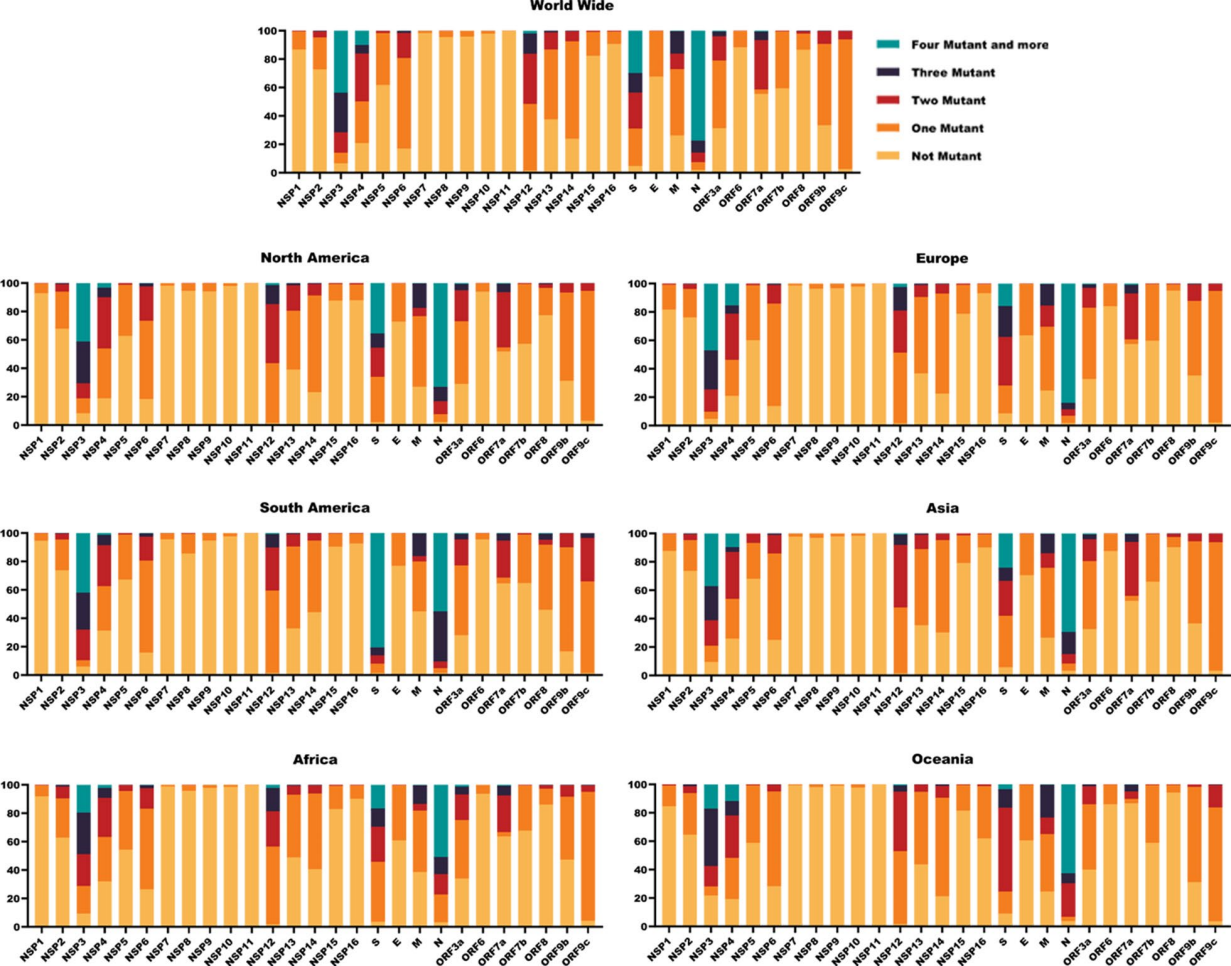
Mutation rate of SARS-CoV-2 genome in different geographic areas. The graph reports SARS-CoV-2 frequency of one, two, three, and four more mutations in the SARS-CoV-2 genome by April 28, 2022, in seven geographic areas

In our Analysis, different SARS-CoV-2 proteins harbor the maximum rate of mutations, including N, nsp3, S, nsp4, nsp12, and M. The rate of four mutations and above is depicted in Fig. 3. We found that the 10.05% of nsp4 protein in SARS-CoV-2 genomes was the rate of 4 mutations and higher (From 1.28% in South America to 15.40% in Europe). Interestingly, our result showed that the average rate of higher than four mutations in SARS-CoV-2 genomes was higher in South America (6.71%) and Europe (6.71%) in comparison to Africa (3.48%) and Oceania (3.54%). Obermeyer et al. revealed that the highest concentrations of fitness-associated mutations were found in the S, N, M, and ORF1 polyprotein genes [56]. NSP4 is the most significant membrane protein of the NSPs, with nsp6 having roles in the assembly of replication compartments. The highest concentration of fitness-associated mutations is found in nsp4, nsp6, and nsp12–14, suggesting new functions at those sites. [56]. Generally, ORF1ab is a conserved region in the SARS-CoV-2 genome [67]. Comparative sequence analysis showed highly conserved sequences within ORF1ab, particularly in nsp12–16 [79]. Robins et al. aligned and compared 149 proteins in β-coronaviruses and found that nsp12–14 are among the most highly conserved in aa identity [66].

In our study, just 2.05% of the world samples did not have any mutation in the N protein. On the other hand, 77.35% of the N protein of SARS-CoV-2 harbor four mutations and above (From 50.76% in Africa to 83.96% in Europe). Our results showed that 43.6% of the nsp3 protein of SARS-CoV-2 harbor four mutations and above (From 17.00% in Oceania to 47.06% in Europe). In the nsp3, 6.61% of worldwide samples had no mutations. NSP3, also known as papain-like protease, the largest NSP, plays a critical role in viral replication and function as a protease. Papain-like protease, polyprotein processing. Type I IFN inhibition is implicated in membrane structure formation induced upon CoV infection, with which the RTC is thought to be associated. SARS-CoV nsp3 is a 215-kDa, transmembrane, glycosylated, multi-domain protein that is a scaffolding protein for these processes [6]. Troyano-Hernáez evaluated the SARS-CoV-2 proteome in Spain and realized that the nsp3 protein has the highest number of deletions and stop codons. However, The PLpro central catalytic residues were highly conserved [82]. Based on fooladinezhad, results, in North America NSP3 data, 41.47% of aa showed more than four mutations in their sequence [22]. The region corresponding to the C-terminal domain of SARS-CoV NSP3 was found to be significantly less mutated likely due to its vital role in inducing the formation of [2] double-membrane vesicles [6]. The most significant number of mutations was located within the gene encoding for the Nsp3 protein (20.7%), followed by the gene encoding for the spike protein (14.6%) [46].

In the S protein, we found that 65.37% of S protein had at least one, two, or three mutations, and 29.79% of S protein in SARS-CoV-2 genomes have a rate of four mutations and higher (From 3.33% in Oceana to 80.52% in South America).

### Conservation in the SARS-CoV-2 genome

**Fig. 4:**
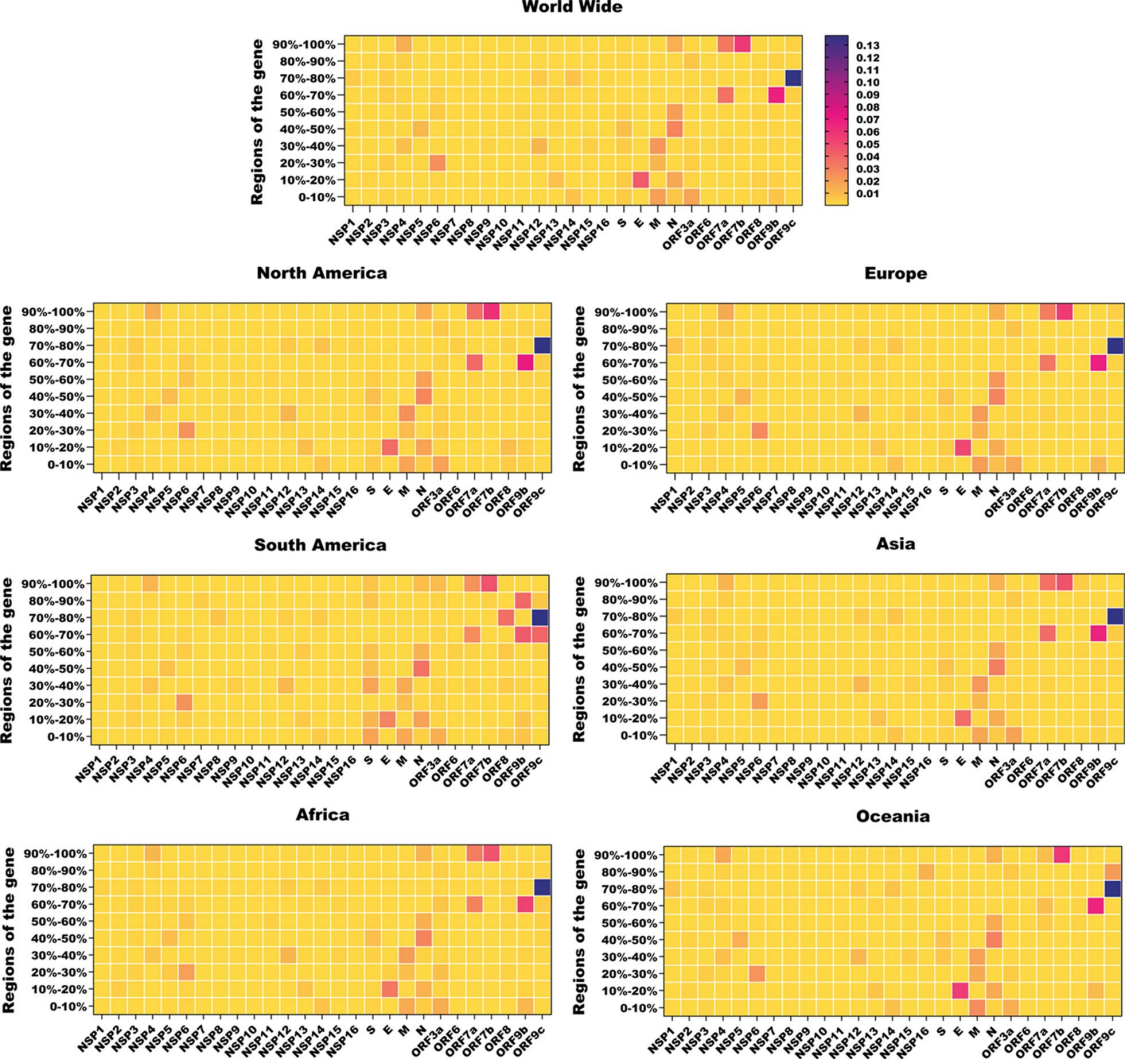
Heat maps of conserved genomic regions of SARS-CoV-2. SARS-CoV-2 genomes are divided into ten regions, and the frequency of mutations is in different regions worldwide. *Nsp* non-structural protein, *S* Spike protein, *E* Envelope protein, *M* Membrane protein, *N* Nucleocapsid protein

We divided each SARS-CoV-2 gene into ten parts, and then we explored the frequency of mutations in each part. In our study, comparative genome analysis between the SARS‐CoV‐2 across Asia, North and South America, Europe, Africa, and Oceania revealed that, on average, the frequency of mutations in NSP10, NSP7, NSP8, NSP11, NSP16, ORF6, NSP9 is lower than 0.040% (Fig. 4). Conversely, the average frequency of mutations was high in ORF9c (1.48%), ORF9b (0.84%), ORF7a (0.74%), and ORF7b (0.59%) (Fig. 4).

To date, different types of tests for SARS-CoV-2 detection-based nucleic acid testing (NAT) have developed, including biosensor chips [3], nanopore targeted sequencing (NTS) [99, 100], multiplex real-time reverse transcription–polymerase chain reaction (rRT-PCR) [30] and ATR-FTIR Spectral Analyses [7]. S protein is considered an essential target for a diagnostic test of SARS-CoV-2, which was evaluated in SARS-CoV and MERS-CoV. In the first months of the pandemic majority of primer/probe sets were designed based on the Wuhan-Hu-1, NC_045512.2 sequences. The United States Food and Drug Administration (USFDA) has authorized 277 SARS-CoV-2 molecular diagnostic tests. Reverse transcriptase polymerase chain reaction (RT-PCR) is the main molecular tests used for SARS-CoV-2. Because of the diverse mutations in the SARS-CoV-2 genome, genetic alternation in the primer binding sites and the probe recognition sites may affect the efficiency of RT-PCR-based detection of COVID-19. Different regions of the SARS-CoV-2 genome, such as the RdRp, S, N,or E genes, are common targets for SARS-CoV-2 by PCR assays [17, 83].

**Fig. 5:**
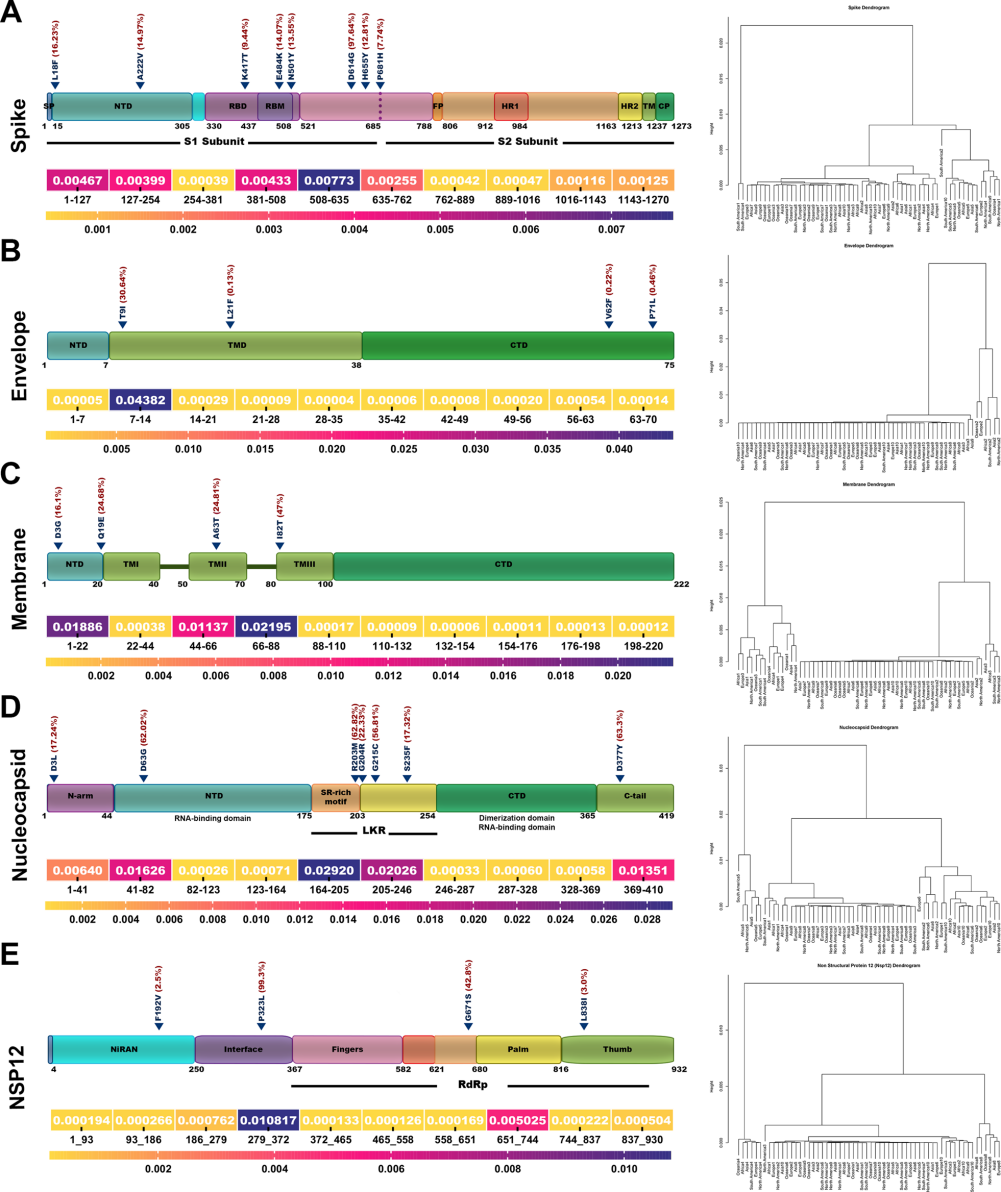
Clustering analysis of SARS-CoV-2 proteins. Heat map and dendrogram illustration of SARS-CoV-2 proteins based on the frequency of mutations in ten different regions of SARS-CoV-2 genomes. **A** Spike. **B** Envelope. **C** Membrane, **D** Nucleocapsid and **E** NSP12. NTD, N-terminal domain; RBD, receptor-binding domain; FP, fusion peptide; HR, heptapeptide repeat sequence; TM, transmembrane; CT, cytoplasmic tail. LKR, serine-arginine (SR) rich-linker region; RBM, receptor binding motif; CP, cytoplasm domain; TMD α-helical transmembrane domain

The average mutation frequency in the structural proteins was 0.027%, 0.045%, 0.53% and 0.088% in S, E, M, and N, respectively (Fig. 5). Furthermore, we have tested whether the frequency of mutations throughout the different regions of S, E, M, N and nsp12 can cluster in different areas including Asia, North and South America, Europe, Africa, and Oceania (Fig. 5).

Conserved regions of the S protein are shown in Fig. 5A which aa 254–381 (0.039%) and aa 726–1016 (0.042%) are conserved. No common mutations were observed in these regions. The regions with the highest mutation frequency in the S were aa 508–635(0.77%) and aa 381–508 (0.43%) Hierarchical clustering results classified the spike protein into the two major clusters. The cluster two sub-classified into two other clusters that one of them contained aa 508–635 that enriched with D614G mutation (Fig. 5A). S protein of SARS-CoV-2 is required to attach and fuse into the host cells to initiate infection. It is the primary target of neutralizing antibodies. The S protein of SARS-CoV-2 is the design basis of different generations of COVID-19 vaccines (Pfizer/BioNTech and Moderna’s). The S protein of SARS-CoV-2 and SARS-CoV share a 76% similarity in the amino acid sequence [14, 15]. The RBD region targets neutralizing antibodies (nAbs), and mutations in the RBD are present in SARS-CoV-2 variants of concern [78]. Previous studies suggested that RBD of CoV in the highly mutable region may not be an ideal drug target [29, 94]. A receptor-binding domain (RBD, 319–541 residues) recognizes the receptor ACE2 specifically. RBD is a critical target for antiviral compounds and antibodies. The most non-conserved region in the S was aa 508–635. Interestingly, region 603–634 of the S protein of SARS is a major immunodominant epitope in S protein [26]. The C662–C671 epitope has also been targeted by neutralizing antibodies [41, 42]. S protein is the well-studied structural protein in the SARS-CoV-2 that mediates human ACE2 receptor binding and is responsible for entry into a cell and endosomal escape. Numerous studies have demonstrated the high frequency of mutations in the spike SARS-CoV2 as variants of concern (VOC) [48]. The study of van Dorp et al. revealed that about 80% of SARS-CoV-2 genome mutations occur in the spike protein [85]. S protein has the highest mean aa change/deletion frequency per sequence in the study of Troyano-Hernáez [82].

In our study, aa 7–14 had the highest frequency of mutation (4.38%) in the E gene. T9I (30.6%) was the most prevalent mutation in the TMD region of the E protein. Hierarchical cluster analysis classified the E protein into two major clusters. The cluster 2 contained aa 7–14 that enriched with T9I mutation (Fig. 5B). SARS-CoV-2 E protein is 228 nucleotides long and has a variety of functions such as viral assembly, replication, propagation, and pathogenesis [64].

In the M protein, the highest mutation frequency was observed in aa 66–88 (2.19%) and aa 1–22 (1.88%). The most common mutations found in this region were I82T (47%), D3G (16.1%), and Q19E (24.68%). M protein classified in two major clusters through a hierarchical clustering. The cluster one sub-classified into two other clusters that enriched aa 1–22 and aa 66–88 that contained D3G, Q19E, and I82T (Fig. 5C). M protein comprises 223 amino acids and performs various functions, including virion formation and assembly. A previous study declared that M protein is conserved across ß-coronaviruses [9]. The sequence identities and sequence similarities between the M proteins sequence of SARS-CoV-2 and SARS-CoV were 90.5% and 96.40%, respectively [50].

In the N protein, the highest frequency of mutation was reported in aa 164–246 ( 2.92%), aa 205–246 (2.02%), and aa 41–48 (1.62%). These regions harbor several common mutations such as R203M (62.82%), D63G( 62.02%), G215C (56.81%), G204R (22.33%), and S223F (17.32%). The result of hierarchical cluster analysis revealed that N protein classified into the three major clusters. The cluster 1 contained aa164-205 that enriched with R203M and G204R mutations (Fig. 5D). The N protein of SARS-CoV-2 has 419 amino acids, with 45.6 kDa positively charged unstable hydrophobic protein, and plays a pivotal role in transcription and replication; interaction with M during viral assembly, N protein is involved in the packing of RNA, the release of virus particles, and the formation of the ribonucleoprotein core. Yu et al. analyzed 5,167,111 N proteins and reported low mutation rates in their amino acid sequences. [96]. In the study of Troyano-Hernáez et al., the mean aa change/deletion frequency per sequence of N was 3.79 [82]. N gene is one of the most non-conserved genes in the SARS-CoV-2 [78]. In the N protein, aa 164–246 was the non-conserved region that harbored common mutations such as R203M, G204R, G215C, and S223F. This region is located in the serine-arginine (SR) rich-linker region (LKR) (aa 175–254) that forms a phosphorylation-dependent binding domain and is responsible for oligomerization, phospho-regulation and RNA and protein binding [13, 91, 93]. It has been reported that the SR-linker was the most variable region within the N protein [81]. It has been reported that R203M, G204R, G215C, and S223F mutations in this region could have an important biological impact and increase the infectivity, fitness, and pathogenicity of SARS-CoV-2 [93]. The N gene of SARS-CoV-2 is the target of many diagnostic assays for COVID-19. Miller et al. reported that two point mutations in the N gene, a C to T mutation at position 29197 and a C to T mutation at position 29200, negatively impact the SARS-CoV-2 detection by the Cepheid Xpert Xpress SARS-CoV-2 assay [52]. N gene mutation C29200T was also reported in Hasan et al. (84) study. The C29197T mutation results in N-gene target failure in the Xpert Omni SARS-CoV-2 assays from Cepheid (Sunnyvale, CA) [47].

In the NSP12 protein, the highest mutation frequency was observed in aa 279–372. The most common mutation P323L (99.3%) in SARS-CoV-2 genomes was observed in this region. The hierarchical cluster analysis classified NSP12 into two major clusters. The cluster 1 contained aa 279–372 that enriched with P323L mutations (Fig. 5E). The SARS-CoV-2 NSP12 is a key component of the viral replication [28]. P323L is a key mutation in the NSP12 and has been associated with a high mutation rate and severity of COVID-19 [11, 58].

### Effect of common SARS-CoV-2 mutations on protein stability

Understanding the stability changes in SARS-CoV-2 proteins is essential for predicting virus infectivity. Furthermore, it could be generating novel insights for updating the COVID vaccines. Changes in Gibbs free energy of unfolding (ΔΔG) between the wild type and mutant protein could predict the effect of the stability of protein structure [59].

**Fig. 6:**
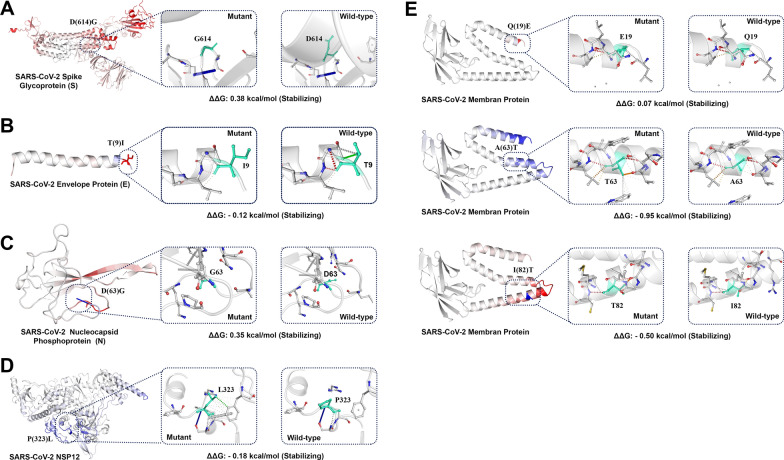
Dynamut prediction of molecular flexibility and destabilizing effect of common SARS-CoV-2 mutations. The protein rigidification and structural flexibility are highlighted in blue and red color, respectively. Light green represents wild-type and mutant residues of protein**. A** Spike mutation D614G**. B** Envelope mutation T9I**. C** Nucleoprotein mutation D63G.** D** NSP12 mutation 9323L**. E** Membrane mutations A63T, I82T and Q19E

In the current study, structured-based analysis results by Dynamut2 and MAESTROweb indicate that the P323L mutation decreases the stability of NSP12. While DynaMut and SDM results showed that, this is a stabilizing mutation (Fig. 6D, Table 2). The topology-based mutation predictor (TML-MP) in the study of Wang et al. suggested that P323L destabilizes the NSP12 [88]. On the other hand, the study of Kim et al. MicroScale Thermophoresis analysis revealed that the NSP12 P323L mutation stabilized the NSP12-NSP7-NSP8 complex interaction [37]. Periwal et al. also suggesetd that the P323L had a stabilizing effect relative to the wild type protein [61].

**Table 2:** Predicted output from DynaMut, Dynamut2, SDM and MAESTROweb for the stability of SARS-CoV-2 proteins

Protein	Mutation	Protein structure	Dynamut	Dynamut2	SDM	MAESTROweb
NSP12	P323L	7C2K	1.532	− 0.18	1.18	− 0.082
Spike	D614G	6VXX	0.299	0.38	2.5	0.101
Envelope	T9I	7K3G	0.214	− 0.12	1.1	2.902
Membrane	A63T	8CTK	0.050	− 0.95	− 1.53	1.351
Membrane	I82T	8CTK	− 0.460	− 0.5	− 1.5	0.485
Membrane	Q19E	8CTK	− 0.120	0.07	0.4	− 0.481
Nucleoprotein	D63G	6VYO	0.007	0.35	0.87	1.101

Our results indicate that all the methods predicted that the Spike D614G and nucleoprotein D63G are stabilizing mutations (Fig. 6A, C, Table 2). The results of Gellenoncourt et al. suggested that the D614G mutation stabilized S1/S2 association and enabled the selection of mutations that increased S1/S2 cleavage [24]. According to DynaMut estimations in the study of Chakraborty et al. D614G is stable mutation [12]. In the study of Aljindan et al., CUPSAT, SDM 2.0,and DUET analysis tools predict increasing stability of the spike protein [4]. Based on the results of FoldX and ROSETTA outputs, Mahmoudi Gomari et al. suggested that the D614G mutation increases the stability of spike protein [49]. Plante et al. measured the decay of infectivity of D614 and G614 viruses over different times and found out the D614G mutation may increase the stability of SARS-CoV-2 [62].

For the membrane protein, Dynamut2 and SDM predict membrane Q19E mutation as a stabilizing mutation. DynaMut and MAESTROweb analysis showed Q19E mutation destabilized structure of the membrane protein. In addition, Dynamut2 and SDM results showed that membrane A63T is destabilizing mutation, while Dynamut and MAESTROweb results indicate that, these are stabilizing mutations. Membrane I82T mutation destabilize protein structure according to Dynamut, Dynamut2 and SDM analysis, however MAESTROweb results showed that this mutation stable membrane structure. (Fig. 6E, Table 2). The Omicron variant of concern is the most mutated SARS-CoV-2 variant (N = 65, including 16 deletions and 3 insertions) and is characterized by several mutations in a membrane such as Q19E, and A63T [5].

Dynamut, SDM and MAESTROweb analysis results indicate that envelope T9I mutation stabilized envelope structure; however, Dynamut2 results showed decreased stability of the encoded proteins (Fig. 6C, Table 2). The T9I is one of the envelope mutations in the Omicron variant, and the DynaMut prediction outcome revealed that this mutation had a stabilizing effect [55, 72]. In support of our in silico findings, the validation of key results using experimental approaches is warranted the impact of changes in protein sequence on protein stability.

## Conclusion

COVID-19 is one of the most significant global health catastrophes, causing more than 6.56 million deaths globally. SARS‐CoV‐2 has a relatively high dynamic mutation rate, and large-scale genome-sequencing efforts have provided a pattern for the global spread and diversification of SARS-CoV-2. Thanks to the GISAID database, we could access viral genomes from all over the world in the study.

Early diagnosis of SARS-CoV-2 infection is essential for controlling and treating COVID-19 patients. WHO recommends RT-PCR and other NAT assays, which are widely applied in different countries. However, these methods’ false-negative results are a significant challenge to controlling the pandemic. Conserved regions in the SARS-CoV-2 genome could be considered promising targets for diagnostic tools and strengthen the detection sensitivity to reduce false-negative results.

Vaccinations resulted in a decline in the risk of COVID-19 infection and hospitalizations worldwide. However, numerous studies have demonstrated that the efficacy of vaccines against infection decreases over time. Therefore, molecular surveillance programs are critical to guide the development of vaccines based on molecular change and novel emerging SARS‐CoV‐2 variants.

## Supplementary Information

**Additional file 1.** The mutation frequency of genes in samples grouped by continents.

**Additional file 2.** Each gene’s length is divided into ten sections, and the frequency of mutations in each region is categorized by continent. These data facilitate the segregation of protected regions from susceptible ones.

## Data Availability

The dataset(s) supporting the conclusions of this article are available in the SARS2Mutant repository, [http://sars2mutant.com/].
